# Interrogating alignment of the 2026 FIFA World Cup event objectives across the Canadian soccer system

**DOI:** 10.3389/fspor.2026.1743648

**Published:** 2026-03-13

**Authors:** Georgia Teare, Kristen A. Morrison, Kerri L. Bodin

**Affiliations:** 1 School of Kinesiology, Western University, London, ON, Canada; 2 Department of Kinesiology, University of Windsor, Windsor, ON, Canada; 3 School of Human Kinetics, University of Ottawa, Ottawa, ON, Canada

**Keywords:** community sport, event impact, event leverage, mega sport event, United26

## Abstract

Both top-down and bottom-up organizations often have event-related objectives associated with hosting major sport events. The extent to which the objectives of these organizations align within the host region can play a role in objective achievement (e.g., through efficient resource mobilization). This study examines the types and alignment of objectives of sport system stakeholders in each of the Canadian host locations of the 2026 FIFA World Cup. A collective case study was employed to interrogate the event-related objectives developed by organizations within the Canadian soccer system. Publicly available documents (*n* = 36 documents; 1,032 total pages) from organizations’ websites were analysed according to their objectives and resources dedicated to support objective achievement. Each organization's objectives were also analysed to interrogate objective alignment across the Canadian soccer system. While some alignment of objectives, particularly among similar types of organizations (i.e., sport, event, non-sport), indicates potential for objective achievement, the lack of clear objectives of some bottom-up organizations may result in missed opportunities. This study contributes to our understanding of event leverage beyond that of sport development and different stakeholder organizations’ tensions in objective alignment.

## 1 Introduction

One-off major sport events are often funded, at least in part, by taxpayer dollars (1). There is therefore an argument to be made that when community members financially contribute to hosting major sport events, there is an obligation for the event to generate benefits for these groups. Over time, event rights holders have increasingly required bid packages to outline intended impacts, including those on the host community, region, and/or nation, resulting from hosting (2). There are a wide range of impacts that have been included in bid documents including, for example, economic benefits, environmental sustainability, sport development, and social advancements. Proponents of major sport events have used the potential for these impacts to justify bringing the event to a host community and to help build community buy-in and support.

When major sport events are awarded to a host nation, region, and community, their potential for impact can be an impetus for a wide range of organizations to use the event to pursue their own organizational objectives (3). These organizations can range from top-down organizations (i.e., stakeholders that control resource allocation) such as local organizing committees and national and regional sport organizations, to bottom-up organizations (i.e., stakeholders that are community-facing) such as community sport organizations (CSOs) (4).

Engagement of top-down stakeholders is important to objective achievement as these groups can control flow of resources and policy implementation. For instance, Liu (5) found that government support was important for legacy impacts of a major sport event. Similarly, Oshimi and Yamaguchi (6) found that top-down stakeholders' engagement with community members was important to objective achievement. These top-down objectives, however, are not always achieved. For instance, Pentifallo and Van Wynsberghe (7) found no evidence of claimed social impacts from the 2010 Vancouver Olympic and Paralympic Games. Top-down stakeholders can also make these claims of impact and state objectives, but without strategic planning and specific tactics to achieve desired outcomes, these objectives are not likely to be met (8) and cost overruns can call into question the necessity of hosting the event in the first place (9).

An often-researched event objective for CSOs is sport development at the grassroots level (10–14). CSOs are grassroots organizations or clubs that provide opportunities for community members to participate in competitive and recreational sprot-related programs (15). While CSOs are therefore necessary to support participation, engagement with top-down stakeholders is needed to ensure there are enough new resources brought into the organization (e.g., facilities, coaching, equipment) to accommodate the new participants (16). In light of this need, CSOs have also attempted to use events to bring in required resources such as volunteers (17, 18). Other community-facing organizations have also developed event-related objectives, such as local businesses and restaurants to take advantage of the increased visitors to the city (19, 20).

It has been theorized (21), and recently empirically supported (4) that strategic alignment between top-down and bottom-up stakeholders can help support event objective achievement due to the streamlining of resources. It has also been found that objectives are less likely to be achieved when there is misalignment between top-down and bottom-up stakeholders (4). When goals are not aligned among stakeholders, leveraging can be difficult to implement and outcomes can be negligible (4, 22). It has been further identified that deliberate partnerships established prior to the event can be effective for developing consensus around goals and implementation tactics (23, 24).

Despite widespread claims and intention to bring about event impacts, there is limited evidence for the realization of these objectives. Most often, these organizations may have event-related objectives but assume that the event simply taking place will lead to desired impact, without any need for strategic planning or specific tactics to elicit desired outcomes (20, 25). However, research has shown that event objectives do not occur on their own – purposeful planning, or leveraging, is needed in conjunction with the event to meet pre-determined objectives (3, 26). The presence of multiple stakeholders with interest in the event and associated outcomes further complicates the process. It is often unclear as to who holds responsibility for generating impact (27). Recent research has emphasized that alignment among top-down and bottom-up stakeholders is necessary for objective achievement (4, 21). In particular, top-down stakeholders are well-positioned to strategically mobilize resources that enable bottom-up organizations within communities to implement leveraging tactics (4). Therefore, the key elements necessary for achieving event-related objectives are the presence of pre-determined goals, and alignment of those goals across stakeholders to ensure coordinated use of resources (i.e., overlap of goal direction).

While alignment is widely acknowledged as important, achieving it in practice remains difficult (28). Further empirical insights are needed to examine the types of objectives stakeholders pursue, if/how resources are mobilized to support these objectives, and how these objectives vary across organizational levels if alignment is (or is not) achieved in major sport event contexts, particularly in the early stages of event planning. The 2026 FIFA World Cup (2026FWC), with multiple Canadian host cities and a complex stakeholder network, presents a valuable context to investigate objective alignment. Thus, this study aims to examine the types and alignment of objectives of sport system stakeholders aligned in each of the Canadian host locations of the 2026FWC. Specifically, three research questions guide this study:

1. What event objectives do various sport system stakeholders adopt with respect to the 2026FWC?
2. How, if at all, are resources being mobilized throughout the Canadian soccer system to achieve objectives?
3. To what extent are the objectives of sport system stakeholders aligned in each of the Canadian host locations of the 2026FWC?

Understanding the types of objectives held by different stakeholders, the extent of alignment between them, as well as resources mobilized, can provide important insights for scholars and practitioners seeking to develop more effective leveraging strategies to optimize event impacts.

This project is underpinned by the *Model for leveraging sport events for sport participation and development* (29). The model was developed via a workshop with 12 experts within the Canadian Sport system who have been, or could be, involved in event leveraging activities. An important element of the model is that there must be goal alignment among focal organizations. While the goals can be driven by any (or all) of the organizations, there must be shared goals. Different types of organizations may have sport participation goals including sport organizations, non-sport organizations, and the event organization.

While the *Model for leveraging sport events for sport participation and development* (29) was developed specifically for sport development, it is helpful for the present study because it provides a framework for examining a sport system and related elements that are applicable to event leverage and multi-stakeholder goal setting. We use this model to identify different stakeholder groups and as a tool of interpreting the goals developed within the context of the Canadian sport system. In doing so, we can interrogate the applicability of the model for event leverage beyond sport development to include the array of event objectives developed by key actors in the 2026FWC.

Sport in Canada is governed through a complex network of organizations at the national, provincial/territorial, and municipal levels (30). National sport organizations (NSOs) are responsible for governing individual sports at the national level including but not limited to developing national team athletes, upholding rules and regulations, and working with provincial and territorial sport organizations to grow participation. Similarly, provincial/territorial sport organizations (referred to in this paper as PSOs) work closely with CSOs which deliver sport participation opportunities to individuals in their communities at all levels. This federated model of sport governance, as seen in Canada, often leads to each member of a sport association developing separate strategic plans, organizational goals, and policies (31). These plans, goals, and policies may not be directly complementary, and indeed may even compete, resulting in a cumbersome sport system (31, 32).

The FIFA Men's World Cup 2026 (2026FWC) is scheduled to be held in 16 cities across the United States, Canada, and Mexico from June 11, 2026 to July 19, 2026 (33). The 2026FWC will host 48 teams, and is the first iteration to be co-hosted by three nations. In Canada, Toronto, Ontario, and Vancouver, British Columbia, are host cities, hosting six and seven matches respectively. Given the complex and multi-level structure of sport in Canada, sport event hosting requires the collaboration and involvement of all levels of government and sport organizations. For example, stakeholders in the 2026FWC governance chart for host city Toronto included the Government of Canada, Government of Ontario, Canada Soccer, Destination Toronto, City of Toronto, as well as venue-specific stakeholders such as Exhibition Place (34).

## 2 Methods

A collective case study methodology (35) was used to interrogate several organizations' goals related to impacts from the 2026FWC. Each organization represents one case, spatially bounded to Canada and conceptually bounded to being a part of the Canadian soccer system and within the two host provinces (Ontario and British Columbia) (36). Each case was examined in-depth to identify objectives (if any) related to the 2026FWC, then the cases were compared to determine objective alignment, if any.

### 2.1 Stakeholder identification

Stakeholders were identified through an iterative document review and stakeholder mapping process. As FIFA awards the rights to host the 2026FWC via a bid procedure, FIFA and the United26 bid committee were our (top-down) event stakeholders. The researchers then conducted a review of the United26 bid book and associated hosting agreements for the 2026FWC, using these documents to iteratively identify additional stakeholder groups explicitly referenced in relation to event planning, governance, and delivery in Canada.

Through this process, the NSO (Canada Soccer) and PSOs (Ontario Soccer, BC Soccer) responsible for soccer governance in host jurisdictions were identified. Similarly, the cities of Toronto and Vancouver were identified as municipal stakeholders responsible for local delivery. Various advocacy groups (e.g., Toronto Board of Trade, Social Change Canada, Greater Vancouver Board of Trade) were also identified.

Community sport organizations were identified as bottom-up sport stakeholders through an iterative search process guided by the publicly identified PSOs. The researchers identified all CSOs located in the host cities (i.e., Greater Toronto Area; GTA and the Metro Vancouver Regional District; MVRD) using the public listing of soccer clubs from the respective PSOs websites. Each CSO's website was then reviewed to identify the presence of publicly available strategic documents. In total, seven CSOs from the GTA and seven CSOs from the MVRD met this criterion and were included in the final sample.

### 2.2 Data collection

Data were collected via publicly available documents from organizations' websites, including key strategic and promotional documents. Table 1 presents the stakeholder group, document type, and number of pages of text analyzed.

**Table 1:** Summary of documents analyzed.

Type of organization	Stakeholder group	Document type	Number of documents (*n* = 36)	Number of pages (*n* = 1,032)
Event (top-down)	FIFA (63–67)	Evaluation reports, industry reports	5	147
	Bid Committee (68)	Bid book	1	530
Non-sport/event (top-down)^a^	Municipalities/organizing committee (69–77)	City council reports, local organizing committee	9	124
Non-sport (bottom-up)	Advocacy groups (78–80)	Municipal board of trade documents	3	47
Sport (top-down)	NSO (81, 82)	Strategic plans, media documents	2	10
	PSOs (83, 84)		2	18
Sport (bottom-up)	CSOs	Strategic plans, strategy documents	14	156

a Due to the co-hosted nature of the 2026FWC, the local organizing committees in Canada are embedded within the local municipalities.

### 2.3 Analysis

Data were analysed via content analysis (37). There are four steps for qualitative content analysis including 1) decontextualization (i.e., identifying meaning units), 2) recontextualization (i.e., review dataset to ensure context is addressed and dross is excluded), 3) categorization (i.e., identification of broad groups), and 4) compilation (i.e., sensemaking) (37). For the present study, the decontextualization stage entailed the identification of event objectives within each case. For example, business development, tourism development, inclusion of specific groups in grassroots programming, and sport infrastructure enhancement are some of the objectives identified. In the recontextualization stage, the authors reviewed the data to ensure objectives were included in analysis, and data not relevant to study aims were excluded. For example, many of the documents included logistical information about planning and implementing the sporting event or objectives related to event implementation rather than impact. This content was deemed not relevant to the study objectives and thus was not included in further analysis. The categorization stage included grouping specific objectives into categories. For example, economic-related objectives were grouped into an “economic development” objectives group, and objectives related to infrastructure refurbishment and access were grouped into a “sport infrastructure” objective group. Finally, for compilation, the authors examined the objective categories across cases to determine the extent to which objectives were aligned (i.e., overlap of goal direction).

## 3 Findings

Table 2 provides a comprehensive overview of findings from the content analysis of publicly available documents related to Canadian soccer system stakeholder's 2026FWC-related objectives. The “Objectives” column addresses research question one (i.e., What event objectives do various sport system stakeholders adopt with respect to the 2026FWC?). The “resource mobilization” column addresses research question two (i.e., How, if at all, are resources being mobilized throughout the Canadian soccer system to achieve objectives?). To address research question three, an elaboration on the alignment between observed objectives follows.

**Table 2:** Overview of findings.

Type of organization	Stakeholder group	Objectives	Resource mobilization	Within stakeholder group alignment
Event (top-down)	FIFA	Promotion of human rights	Required human rights reports from each potential host city	No clear alignment
	Bid Committee	Team Base Camps and infrastructure Sport development for players, officials, and coaches to encourage healthy active lifestyles, and life-long participation for all. Innovating the men's game by aligning the current high-performance structure across all age groups	N/A	
Non-sport/event (top-down)^a^	Municipalities/organizing committee	Vancouver: none stated Toronto: environmental sustainability, supporting local businesses	Vancouver: community activation playbook, licencing opportunities, resources for businesses Toronto: funding infrastructure projects; community activation toolkit; community activation fund	Alignment (not all objectives/ organizations)
Non-sport (bottom-up)	Advocacy groups	Supporting local businesses	N/A	
Sport (top-down)	NSO	Work with provinces to capitalize on the domestic attention brought by the event Facility investment, specifically for domestic Base Camps Sponsorship opportunities for women's soccer Referee development in remote geographic regions Diversify revenues through a foundation and donations program Expand the online learning platform Explore extending associate membership opportunities to ensure adherence to the Safe Sport Roster Invest in digital platforms to raise awareness of high-performance teams Digital infrastructure for coach education, club development, and referee training Participation in international competition Develop programming and acknowledgement for volunteers	‘Club+’, resources for CSO including “tools, templates, and strategies to help grow the game”	Alignment (not all objectives)
	PSOs	BC: 1) Encourage engagement with FIFA26 across the province 2) Drive participation and program development ON: No objectives related to FIFA	BC: event volunteer program ON: marketing activations; branding resources available for members	
Sport (bottom-up)	CSOs	Not explicit in publicly available documents	N/A	N/A

a Due to the co-hosted nature of the 2026FWC, the local organizing committees in Canada are embedded within the local municipalities.

Of note, there is no explicit alignment between FIFA as an organization and the objectives stated in the United26 bid book. While the city of Vancouver documents do not have stated event objectives, there is alignment in the city of Toronto documents around supporting local businesses. While there is not explicit direct alignment for all objectives in publicly available documents among the NSO (Canada Soccer) and the PSOs (BC Soccer and Ontario Soccer), there is some overlap around increasing participants and engagement with the event between the BC PSO and the NSO. The Ontario PSO has a well-developed strategic plan; however, it does not explicitly include a role for FIFA. Finally, FIFA is not explicitly mentioned in the strategic planning documents of the sampled CSOs.

When examining objectives across stakeholder and stakeholder groups, it is evident that FIFA's explicit objectives, as determined through analyzing FIFA industry reports and evaluation reports, are not reflected within the Canadian soccer system. Some of the objectives outlined by the United26 bid committee in the bid book align with broader objectives in the Canadian soccer system, particularly around sport development through elements such as coach and referee support, as seen in the NSO objectives. A difference between the bid and the Canadian sport organization objectives is the target group for sport development support: the bid places an emphasis on growing the men's game, while the NSO is more focused on developing the women's game.

The municipalities' publicly available objectives do not align with the sport-specific organizations' objectives. This could present some issues as in this context, the municipalities have a dual role of local organizing committee. These findings are discussed in the following section.

## 4 Discussion

Through an analysis of publicly available documents of Canadian soccer system stakeholders and event-related organizations, this study examines the types and alignment of objectives of sport system stakeholders in each of the Canadian host locations of the 2026FWC. The following sections discuss and elaborate on the findings above in relation to Chalip's (29) model.

### 4.1 Types of organizations

#### 4.1.1 Event related organizations

In the context of the 2026FWC, the key event-related organizations include FIFA, the Bid Committee, and the municipalities where venues are located (acting as the local organizing committee). Among the event-related organizations, there is not much alignment of publicly communicated event objectives. FIFA's only stated objective is to support human rights, while the bid states objectives related to (men's) sport development, and the municipalities are focused on supporting local businesses. This finding raises the question of what the event rights holder's role should be in developing and supporting event objectives in terms of impact on the host city's sport development. When it comes to event impacts, the question of ownership often arises (29). As each country will have different objectives in terms of sport development, expecting the event rights holder to continually develop new event objectives to align with their host cities seems counter-intuitive to strategic planning. On the other hand, as each country will have their own event-related objectives, expecting the event rights holder to only select countries whose objectives align with their own may be limiting the pool of potential applicants. With the rise of joint bids to co-host major sport events across multiple countries (38–40), the plausibility of the rights holder organization's goals aligning with all host nations is precarious. In the present study, Canada, Mexico, and the United States all had their own unique objectives related to hosting the event (41). The expectation that FIFA would align with all the different objectives is not realistic. Perhaps from this case, we can learn that the rights holder should focus on event implementation and only one very specific and defined goal to mandate from host cities. While it can be argued that FIFA's focus on human rights is performative, each host city was required to conduct a human rights evaluation and submit a report. By having only one requirement in terms of social impact, there were clear outcomes and tangible deliverables expected of host cities.

#### 4.1.2 Sport related organizations

The sport-related organizations include the NSO (top-down), the PSOs in Ontario and British Columbia (top-down), and CSOs in the GTA and MVRD (bottom-up). The NSO has a wide range of event objectives, primarily focused on supporting high-performance sport. British Columbia has a much narrower scope that balances high performance and grassroots sport. This delineation of foci aligns with the organizations' typical purviews: NSOs support high-performance sport in the country and contribute to overall sport system development, PSOs focus on high performance pathways in their province and sport development, while CSOs focus on grassroots participation (42, 43). The British Columbia PSO's few goals align with those of the NSO, which is promising for objective achievement in that province. However, while the Ontario PSO has several objectives in their strategic plan, none of which are explicitly linked to FIFA, it is possible that additional objectives related to the event exist internally but are not publicly documented. Without a clear publicly stated strategic direction related to the event, leveraging efforts may not receive priority.

None of the CSOs in either province have explicit reference to FIFA in their strategic planning material, which can limit 2026FWC event leveraging efforts. This is perhaps not surprising; although CSOs are increasingly becoming formalized and professionalized (44), previous research has suggested that strategic and long-term planning can be challenging for these organizations (45–47). While some CSOs have formal, written strategic plans (48), many do not. Instead, any planning many CSOs undertake tends to be short-term and focused on sport-specific elements, such as coach training (46, 49, 50). As such, CSOs may not have the planning capacity to consider and formalize long-term implications of major events, such as the World Cup, in a written strategic plan. Alternatively, they may choose to focus on factors in their environment that they perceive to be in their immediate control (i.e., sport-specific programs) rather than rely on communication and/or resources from other organizations in the sport system to provide direction with respect to major events (e.g., NSOs, PSOs). This localized focus may be further reinforced by unclear guidance from top-down stakeholders or inconsistent engagement in early planning stages. It is possible that CSOs may try to undertake some form of leveraging initiatives as the 2026FWC approaches, however, reactive or last-minute planning may limit their ability to access external support, build partnerships, or align their activities with event objectives.

While some of the sport development goals seem to be aligned, one area of misalignment between NSO and PSO objectives is around their publicly communicated goals related to women and girls' participation and development. The stated goals around development of the women's game could indicate that women and girls are not participating at desirable rates. There are many well-known reasons for a lack of girls' participation in sport, largely indicating that “traditional” forms and systems of sport participation are not effective to reaching these target audiences (51–53). There needs to be targeted efforts to create programs and club environments that better meet the needs of the audience, which requires deliberate planning and resources.

#### 4.1.3 Non-sport organizations

The non-sport organizations include the municipality (top-down) and local advocacy groups (bottom-up). These organizations have publicly communicated objectives around supporting local businesses, which are aligned. The municipal documents are focused on logistics of hosting, or economic outcomes of hosting, which makes sense given that the municipality is also tasked with managing the local staging of the event. The local non-sport organizations are largely advocacy groups for local businesses. The local municipality would typically be considered a non-sport organization, but because in this case they are taking on the planning of the event, they blur the lines of the sport and non-sport organizations. Beyond the event, the municipalities are clearly non-sport organizations, which is reflected in their stated event objectives being not sport related.

### 4.2 Goal alignment between organization types

An integral component of Chalip's (29) model is that there must be goal alignment among the event stakeholder groups. As noted above, FIFA and the Canadian sport system's goals are not aligned. When examining alignment between the remaining event-related organizations, sport organizations, and non-sport organizations, there is less alignment in their publicly communicated objectives. The event-related organizations are focused on sport development (of the men's game) and economic impact. The focus here on the men's game likely reflects the gendered nature of the event and may reflect the need to develop Canada's elite men's soccer development relative to the development of the country's elite women's program. In contrast, the sport organizations are focused on multiple facets of sport development (more so for the women's game), which likely reflects the need to better support women and girls' sport participation in general. The non-sport organizations are focused on economic impacts. This misalignment raises concerns about the tensions between economic impact and sport development. Many of the resource-controlling organizations have goals related to the business communities and economic impact, leaving the resources necessary for sport development impacts questionable. With increasing costs of hosting and exorbitant cost overruns of major sport events documented (54), event providers have begun more heavily relying on the notion of positive social impacts to support hosting efforts, including sport development and sport participation impacts. For example, in the lead-up to the London 2012 Olympic and Paralympic Games, there was a clear message and goal that hosting the Games would increase sport participation in London and the host country (55). However, scholars found that despite this broad stated objective, limited increase of sport participation levels in the area have been observed (55–57). Furthermore, researchers have noted that in resource-lacking, or austere, contexts, participation outcomes from sport events are challenging to achieve (58). Thus, dedicating targeted resources to sport participation objectives is necessary when considering achieving event-related objectives. Based on the documents analyzed in this study, which only capture formalized and publicly available objectives, a lack of alignment between resource-controlling organizations and bottom-up organizations is observed. However, this does not necessarily imply that bottom-up organizations do not have any sport development objectives, but rather these objectives may not be formalized in a way that is visible and aligned with top-down organizations.

### 4.3 Limitations and implications

As this study relies on official organizational publicly available documents, it is important to recognize that these texts tend to focus on external communication and legitimation. In addition, many of the CSO sample did not have formal documents available online. Thus, the documents used in this study may not fully capture the extent of organizations' objectives around the 2026FWC. Future studies could therefore extend this work by incorporating qualitative methods, such as focus groups or interviews, to examine how organizational priorities related to the 2026FWC are discussed, negotiated, and understood internally. Such approaches may support researchers in moving beyond publicly articulated narratives to enhance understanding of whether or how divergences emerge between externally communicated objectives and organizational priorities related to the 2026FWC.

Practically, this project also identifies that among similar types of organizations (i.e., sport, event, non-sport), objectives can be fairly aligned. However, objectives between types of organizations remain less aligned, which is a consistent issue identified in event leveraging literature (14, 21, 59). For instance, the failure of the 2012 London Olympic and Paralympic Games to inspire new sport participation among British residents has been attributed to a lack of top-down (e.g., the event organizing committee, Sport UK) and bottom-up (e.g., local sport organizations) stakeholder alignment (12, 60–62). Some mechanisms for strengthening alignment can include engagement with community sport clubs well in advance of the event (60), top-down stakeholders engagement with media to help communicate their intentions (12), inclusion of local community members' voices in the objective development process (62), and the creation of formalized collaborative groups with membership from a variety of stakeholder groups (23).

## Data Availability

The original contributions presented in the study are included in the article/Appendix 1, further inquiries can be directed to the corresponding author.

## Appendix A

**Table A1:** List of documents included in analysis.

Type of organization	Stakeholder group	Document	Year of publication
Event (top-down)	FIFA	Human Rights Submissions FIFA World Cup 2026^TM^ Evaluations of FIFA World Cup Host City Stakeholder and Human Rights Submissions: Summary Report The Promise of a Positive Legacy: The 2026 FIFA World Cup Host City Candidates’ Human Rights Plans Update on FIFA's Human Rights Due Diligence for the FIFA World Cup 2026	2022 2022 2022 2022 2022
	Bid Committee	United 2026 Bid Book	2018
Non-sport/event (top-down)	City of Toronto City of Vancouver	FIFA World Cup 2026 Candidate Host City Human Rights Stakeholder and Partner Engagement Report Evaluation of Toronto's Human Rights Stakeholder Engagement Submission to FIFA Proposed Business Plan for Toronto's Participation in FIFA 2026 Update on Hosting FIFA World Cup 2026 Update on Hosting FIFA World Cup 2026 FIFA World Cup 2026 Toronto Governance, Community Benefits Plan, Legacy and Program Advisory Framework, FIFA Fan Festival Kicking Off Community Benefits for the 2026 FIFA World Cup 26 Vancouver Website: ‘Commercial Opportunities’ page	June 2021 July 2022 March 2023 March 2022 February 2024 July 2024 April 2024 No date
Non-sport (bottom-up)	Toronto Board of Trade Buy Social Canada Greater Vancouver Board of Trade	Ripple Effect: Unlocking Toronto's Waterfront Potential Ensuring FIFA World Cup 2026 Creates Economic and Social Value in Toronto Vancouver's Event Horizon with Ken Cretney and Chris May – Hosting Rights Episode 2	May 2024 No date
Sport (top-down)	Canada Soccer	2022–2026 Canada Soccer Strategic Plan FIFA Announces Vancouver and Toronto as Canadian Host Cities for the FIFA World Cup 2026	No date June 2022
	PSOs	Ontario Soccer Strategic Plan 2022–2026 BC Soccer Strategic Plan 2025–2028	2022 No date
Sport (bottom-up)	CSOs	Strategic plan and strategy documents of seven CSOs in the MVRD and seven CSOs in the GTA	
